# Distinct and Overlapping Roles for AP-1 and GGAs Revealed by the “Knocksideways” System

**DOI:** 10.1016/j.cub.2012.07.012

**Published:** 2012-09-25

**Authors:** Jennifer Hirst, Georg H.H. Borner, Robin Antrobus, Andrew A. Peden, Nicola A. Hodson, Daniela A. Sahlender, Margaret S. Robinson

**Affiliations:** 1 University of Cambridge, Cambridge Institute for Medical Research, Cambridge CB2 0XY, UK

## Abstract

Although adaptor protein complex 1 (AP-1) and Golgi-localized, γ ear-containing, ADP-ribosylation factor-binding proteins (GGAs) are both adaptors for clathrin-mediated intracellular trafficking, the pathways they mediate and their relationship to each other remain open questions [1]. To tease apart the functions of AP-1 and GGAs, we rapidly inactivated each adaptor using the “knocksideways” system [2] and then compared the protein composition of clathrin-coated vesicle (CCV) fractions from control and knocksideways cells. The AP-1 knocksideways resulted in a dramatic and unexpected loss of GGA2 from CCVs. Over 30 other peripheral membrane proteins and over 30 transmembrane proteins were also depleted, including several mutated in genetic disorders, indicating that AP-1 acts as a linchpin for intracellular CCV formation. In contrast, the GGA2 knocksideways affected only lysosomal hydrolases and their receptors. We propose that there are at least two populations of intracellular CCVs: one containing both GGAs and AP-1 for anterograde trafficking and another containing AP-1 for retrograde trafficking. Our study shows that knocksideways and proteomics are a powerful combination for investigating protein function, which can potentially be used on many different types of proteins.

## Graphical abstract

**Graphical Abstract:**

**Figure:**
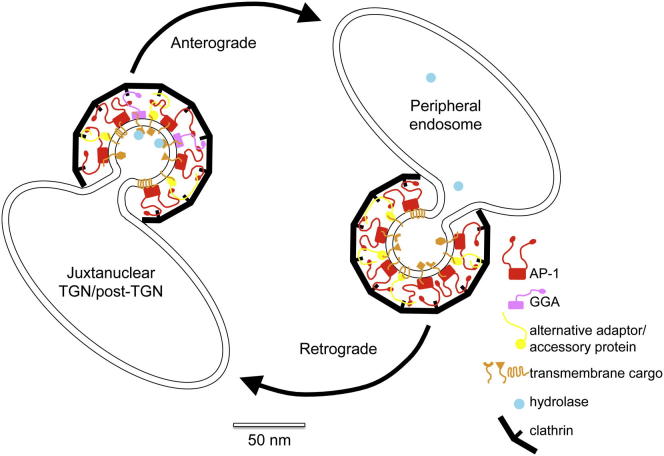

**Highlights:**

► AP-1 knocksideways depletes ∼100 proteins from clathrin-coated vesicles (CCVs) ► GGA2 knocksideways mainly depletes hydrolases and their receptors ► GGA2 depends on AP-1 for incorporation into CCVs ► AP-1 acts as a linchpin for intracellular CCV formation and is bidirectional

## Results and Discussion

### Rerouting of AP-1 and GGA Adaptors to Mitochondria

The clathrin adaptor AP-1 (adaptor protein complex 1) is expressed in all eukaryotes and has been implicated in pathways as diverse as sorting of lysosomal hydrolases and/or their receptors in mammals [3, 4], protein trafficking to olfactory cilia in *C. elegans* [5], biogenesis of rhoptry organelles in Toxoplasma [6], formation of contractile vacuoles in Dictyostelium [7], and protein localization to the *trans*-Golgi network (TGN) in yeast [8]. However, exactly what AP-1 is doing is still unclear, including whether it facilitates anterograde trafficking, retrograde trafficking, or both. Its relationship to the GGA (Golgi-localized, γ ear-containing, ADP-ribosylation factor-binding) family of adaptors is also a topic of much speculation [9–11]. Although knockdowns and knockouts have provided some insights into AP-1 and GGA function, they have also yielded conflicting results [3, 4, 8, 9, 11] and/or surprisingly subtle phenotypes [10, 12]. This is most likely because knockdowns and knockouts take a long time, during which the cell can adjust to the gradual loss of the protein of interest by switching on compensatory pathways.

**Figure 1:**
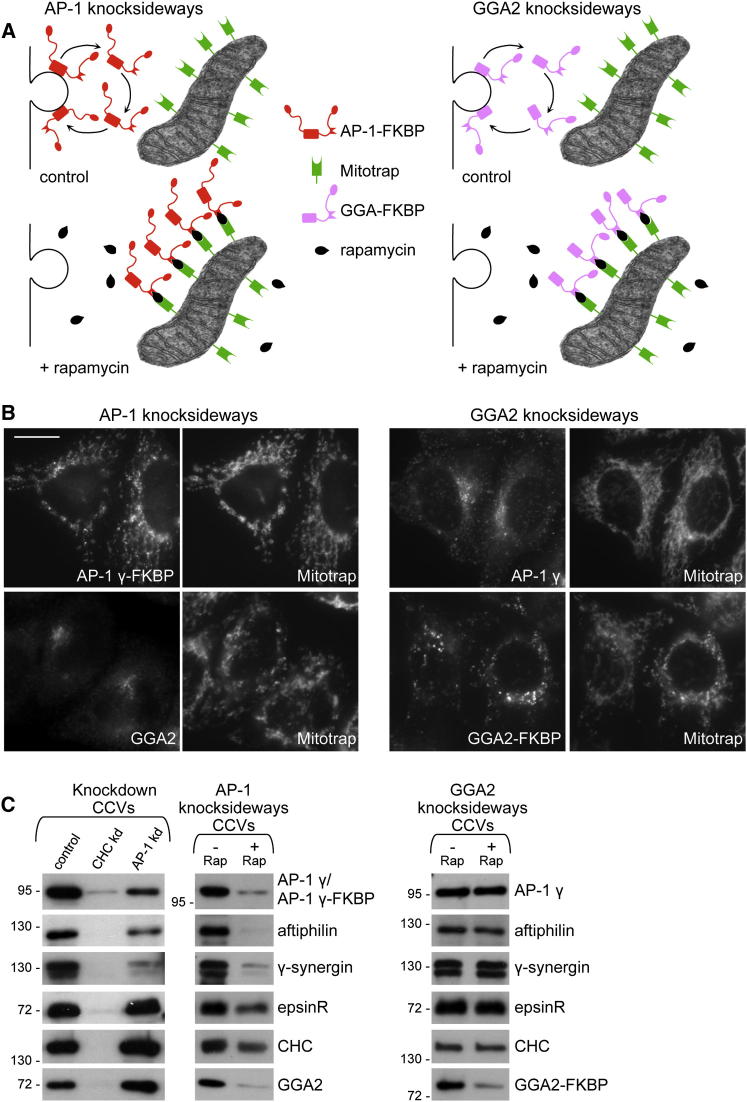
Knocksideways of AP-1 and GGA2 (A) Schematic diagram of the AP-1 and GGA2 knocksideways. (B) Immunofluorescence double labeling of cells after an AP-1 or GGA2 knocksideways. In both cases the FKBP-tagged adaptor was rerouted to mitochondria, but GGA2 did not follow AP-1 onto mitochondria, nor did AP-1 follow GGA2. Scale bar represents 20 μm. (C) CCV fractions after either a conventional clathrin heavy chain (CHC) or AP-1 knockdown (kd) (far left), an AP-1 knocksideways (left), or a GGA2 knocksideways (right). The conventional clathrin knockdown caused a dramatic loss of other coat components; the AP-1 knockdown had a weaker effect on aftiphilin and γ-synergin, and no effect at all on epsinR, clathrin, or GGA2; and the AP-1 knocksideways caused all of these coat proteins to be depleted. The GGA2 knocksideways caused GGA2 itself to be lost from CCVs, but other coat proteins were unaffected. See also Figure S1.

We recently developed a technique called a “knocksideways” to circumvent the problem of gradual protein loss [2]. The knocksideways technique involves tagging a protein of interest with an FKBP domain, making it siRNA (small interfering RNA) resistant, and then coexpressing it with a protein called Mitotrap, which is tagged with an FKBP-rapamycin-binding domain and anchored into the mitochondrial outer membrane. The endogenous protein of interest is knocked down with siRNA, and rapamycin is added to the cells. Rapamycin sequesters the protein of interest onto mitochondria by causing it to form heterodimers with Mitotrap, thus depleting it from the available cytosolic pool (Figure 1A). In the case of AP-1, rerouting to mitochondria is essentially complete by 10 min, so the knocksideways is ∼500 times faster than a conventional knockdown [2].

To investigate the effect of the AP-1 knocksideways on some of AP-1's binding partners, we first carried out western blots on isolated clathrin-coated vesicles (CCVs). Addition of rapamycin for 10 min caused γ-FKBP to be strongly depleted from CCVs, similar to endogenous γ in a conventional AP-1 knockdown (Figures 1B and 1C, left). However, the conventional knockdown had a relatively weak effect on the AP-1 accessory proteins aftiphilin and γ-synergin and no apparent effect on epsinR. In contrast, all three of these proteins were substantially depleted from CCVs isolated from AP-1 knocksideways cells, and we also saw a partial loss of clathrin, as previously reported [2] (Figure 1C; see Figure S1 available online). Most unexpectedly, we found that GGA2 (one of three GGAs in mammals and the only one detectable in isolated HeLa cell CCVs by either western blotting or mass spectrometry) was strongly depleted, even though GGA2 did not follow AP-1 onto mitochondria (Figure 1B, left). In contrast, in a conventional AP-1 knockdown, the amount of GGA2 in the CCV fraction was unaffected (see also [10]). Thus, the knocksideways is much more effective than a knockdown at revealing which proteins normally depend upon AP-1 for their incorporation into CCVs, and it uncovers an unexpected relationship between AP-1 and GGA2.

To tease apart the roles of AP-1 and GGAs, we made a second cell line coexpressing Mitotrap and a siRNA-resistant GGA2-FKBP construct (Figures 1A and 1B, right). Because the mammalian GGAs are thought to be at least partially redundant [13–15], all three endogenous GGAs were depleted with siRNA. Addition of rapamycin for 10 min caused the GGA2-FKBP to become rerouted to mitochondria and lost from isolated CCVs (Figures 1B and 1C). However, western blotting did not show a concomitant loss of any other coat components from the CCV fraction. Taken together, the AP-1 and GGA2 knocksideways phenotypes indicate that GGA2 depends on AP-1 for incorporation into CCVs, but not vice versa.

### Profiling of CCVs from AP-1 and GGA Knocksideways Cells

To look for changes in the overall protein composition of CCVs in cells with either AP-1 or GGA2 rerouted to mitochondria, we used SILAC (stable isotope labeling by amino acids in cell culture) followed by mass spectrometry. Endogenous AP-1 or GGAs were depleted using siRNA, and the SILAC-labeled cells were treated for 10 min with rapamycin before the initial homogenization step. CCV fractions were prepared from cells treated both with and without rapamycin, and the relative abundance of each protein was quantified (Table S1).

**Figure 2:**
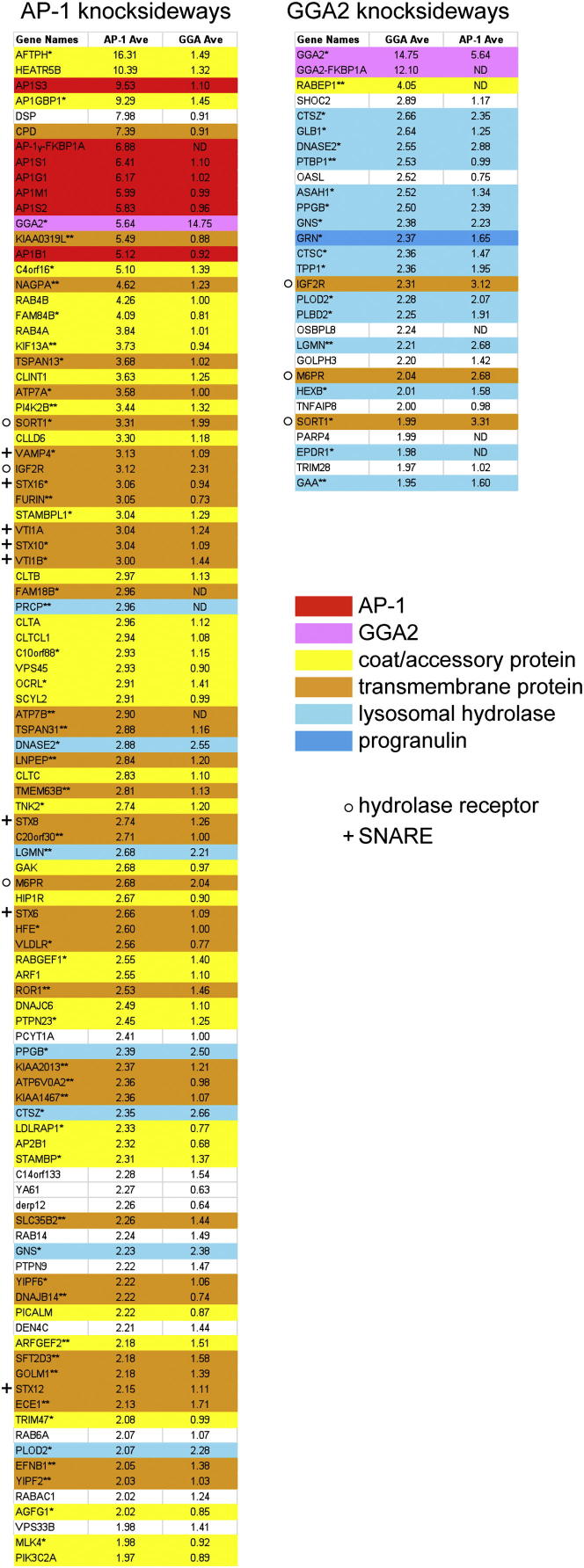
Proteomics of CCVs from SILAC-Labeled Knocksideways Cells The fold change (control:knocksideways) was calculated for each protein, and proteins were ranked from the highest to lowest ratio. Proteins with ratios of 2.0 or higher are shown in order of rank for both the AP-1 and GGA knocksideways, with color coding for those that were previously identified as likely coat/accessory proteins, and for transmembrane and lysosomal lumen proteins. Proteins marked with a double asterisk were identified as likely CCV components by proteomics for the first time in the present study; those marked with a single asterisk were identified as likely CCV components in our recent profiling study [17], but not in previous proteomics studies. The most striking difference between the AP-1 and GGA knocksideways is the preponderance of lysosomal hydrolases in the GGA2 knocksideways, whereas the AP-1 knocksideways affected a wide range of proteins. See also Table S1.

Figure 2 shows that 99 proteins were reduced 2-fold or more in the AP-1 knocksideways, compared with only 29 proteins in the GGA2 knocksideways. In addition, the AP-1 and GGA knocksideways had strikingly different effects on different types of proteins. In the AP-1 knocksideways, the most strongly depleted proteins were mainly other coat components or accessory factors. These include GGA2, which was depleted 5.6-fold in the AP-1 knocksideways. A total of 37 transmembrane proteins were robustly affected, including 8 SNAREs (soluble N-ethylmaleimide-sensitive factor-activating protein receptors), and 7 lysosomal hydrolases were also depleted.

In the GGA2 knocksideways CCV fraction, only one other known accessory protein was significantly lost: rabaptin-5 (RABEP1), which interacts with both the appendage and the GAT domain of GGAs [16]. Hydrolases and other lysosomal lumen proteins (e.g., progranulin) were the major class of affected proteins, with 16 showing losses of 2-fold or more. Only three transmembrane proteins were depleted 2-fold or more, and these were the three hydrolase receptors: the cation-independent and cation-dependent mannose 6-phosphate receptors (IGF2R and M6PR) and sortilin (SORT1).

### Hydrolases and Their Receptors

**Figure 3:**
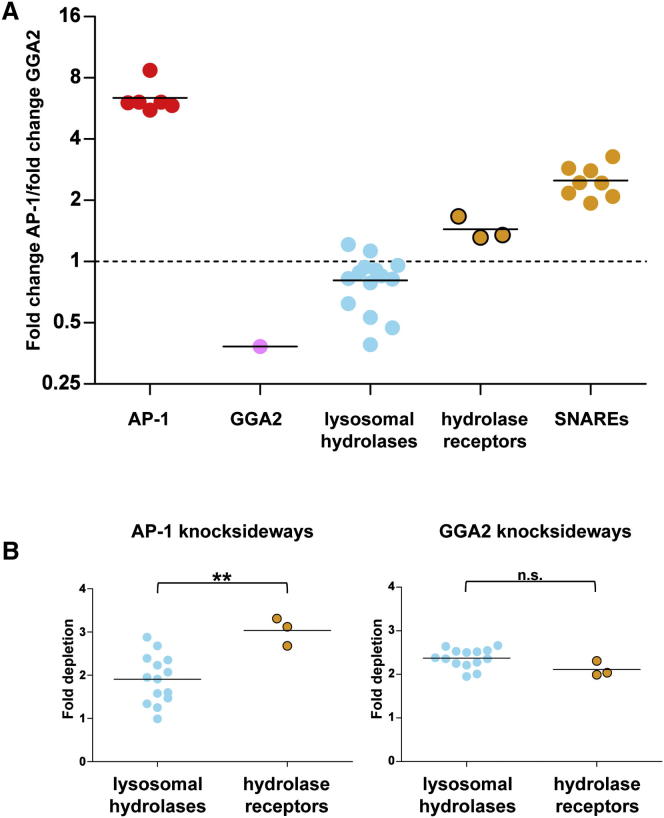
Comparison of the AP-1 and GGA2 Knocksideways (A) Average depletion in the AP-1 knocksideways divided by the average depletion in the GGA2 knocksideways, shown for selected CCV components. (B) The GGA2 knocksideways depleted hydrolases and sorting receptors equally, whereas the AP-1 knocksideways depleted the hydrolase receptors more strongly (^∗∗^p < 0.01). n.s., not significant. See also Figure S2.

To compare the effects of the AP-1 and GGA2 knocksideways on different types of proteins, we plotted the average depletion in the AP-1 knocksideways divided by the average depletion in the GGA2 knocksideways. Figure 3A shows these data for the adaptors themselves, for all of the hydrolases and/or lysosomal lumen proteins that were depleted at least 2-fold, for the three hydrolase receptors, and for all of the SNAREs that were depleted at least 2-fold. The hydrolases stand out as the only proteins, other than GGA2 itself, that were at least as strongly depleted in the GGA2 knocksideways as in the AP-1 knocksideways. This result indicates that the principal function of GGA2 in HeLa cells is to traffic hydrolase-receptor complexes in the anterograde direction.

The role of AP-1 in hydrolase trafficking is more difficult to dissect. The hydrolases were depleted nearly as strongly in the AP-1 knocksideways as in the GGA2 knocksideways (Figures 3A and 3B); however, because the AP-1 knocksideways also depleted GGA2, it seems likely that hydrolases are trafficked mainly (if not entirely) by GGAs. In contrast, whereas the GGA2 knocksideways depleted hydrolases and their receptors equally strongly, the AP-1 knocksideways depleted the receptors significantly more than the hydrolases (Figure 3B). This indicates that some of the AP-1 is involved in the trafficking of ligand-free receptors, most likely in the retrograde direction.

### Coat Components and Accessory Factors

Other proteins affected by the AP-1 and/or GGA2 knocksideways include a number of peripheral membrane proteins that have been shown to bind in vitro to the γ and/or GGA C-terminal appendage domains, as well as other peripheral membrane proteins predicted to be novel CCV components [17] (Table S1). Among the most strongly affected peripheral membrane proteins were Rab4a and Rab4b. Historically, Rab4 is thought to be endosome associated and AP-1 to be TGN associated, but it is now clear that the two organelles are closely interlinked, both structurally and functionally, and often difficult to distinguish from each other. Indeed, a recent study proposed that in plant cells the TGN doubles as an early endosomal compartment, and the same may be true for animal cells [18]. Another small guanosine triphosphatase, ADP-ribosylation factor 1 (ARF1), which facilitates both AP-1 and GGA recruitment onto membranes, was also depleted in AP-1 knocksideways CCVs, as were exchange factors for both Rab4 (RABGEF1) and ARF1 (ARFGEF2).

### Integral Membrane Proteins

Although the only integral membrane proteins depleted 2-fold or more by the GGA2 knocksideways were the three hydrolase receptors, the AP-1 knocksideways had strong effects on 37 different integral membrane proteins, including carboxypeptidase D and furin, two proteases that cycle between the TGN and endosomes using acidic clusters as sorting signals. PACS-1 (phosphofurin acidic cluster-sorting protein 1) has been proposed to link acidic cluster-containing proteins to AP-1 [19]; however, we found only trace amounts of PACS-1 in our CCV fraction, and it did not change in the AP-1 knocksideways, indicating that PACS-1 is unlikely to be the acidic cluster adaptor.

The largest group of integral membrane proteins to be affected by the AP-1 knocksideways was the SNARE family. SNAREs are essential vesicle cargo proteins, because without them vesicles would be unable to fuse with their targets, but with the exception of VAMP4 (vesicle-associated membrane protein 4), which has a dileucine motif that binds to AP-1 [20], most SNAREs lack conventional sorting signals. VAMP4 was the only R-SNARE to be robustly affected, but several Q-SNAREs were depleted, including syntaxins 16, 10, 8, 6, and 12, and Vti1 a and b. Vti1b has been shown to use a folded domain to interact with epsinR/clathrin interactor 1 [21], an AP-1-associated protein that was also strongly affected by the knocksideways. Whether the other SNAREs can bind directly to AP-1 or another coat protein or whether they bind indirectly via VAMP4 and/or Vti1b remains to be determined.

Other strongly depleted transmembrane proteins include several that are mutated in human genetic disorders (http://omim.org). KIAA0319L and NAGPA, two proteins that traffic in a clathrin-dependent manner, have been implicated in dyslexia and persistent stuttering, respectively [22–24], whereas the copper-transporting ATPases (adenosine triphosphatases), ATP7A and ATP7B, are mutated in two disorders of copper metabolism, Menkes disease and Wilson disease. Under normal conditions, the two copper-ATPases are found mainly in the TGN, but they translocate to the plasma membrane in response to high copper levels, and both have dileucine motifs that contribute to their TGN localization [25].

**Figure 4:**
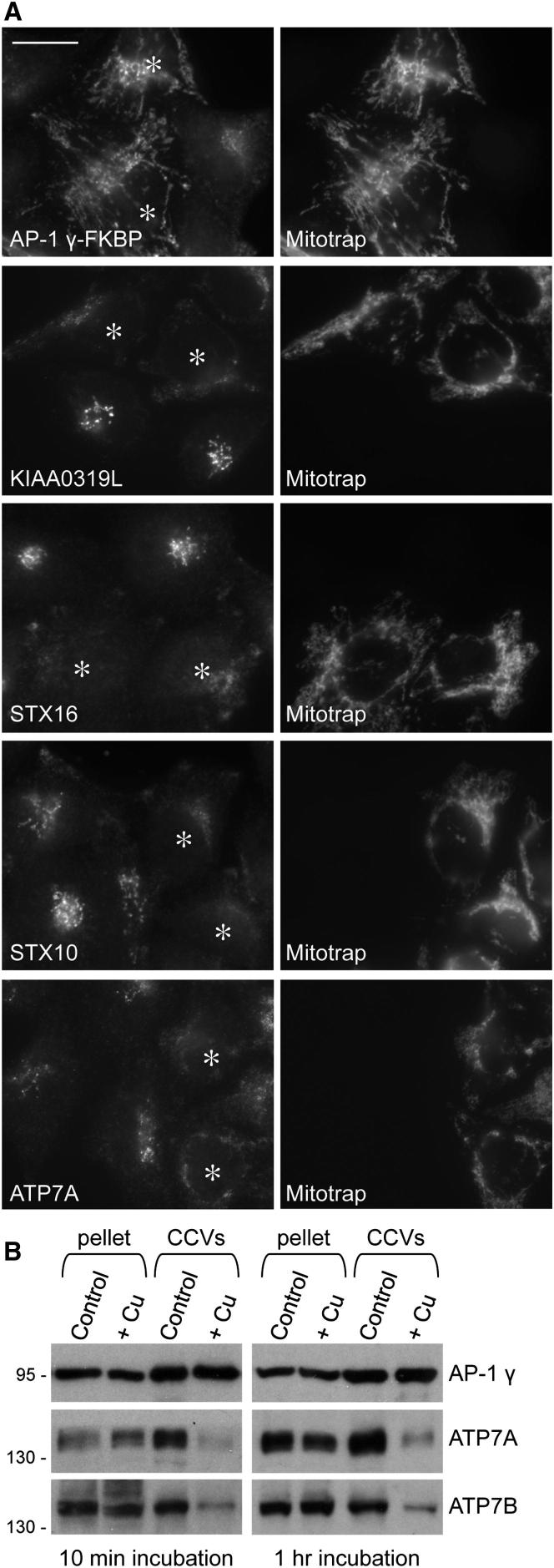
Sorting of Selected Cargo Proteins (A) Cells expressing either FKBP-tagged AP-1 γ alone or FKBP-tagged AP-1 γ plus Mitotrap were mixed together, depleted of endogenous AP-1 γ, and treated with rapamycin for 1 hr, then double labeled for immunofluorescence. In the Mitotrap-expressing cells (asterisks), rapamycin caused the cargo protein labeling to become more diffuse and peripheral. Scale bar represents 20 μm. See also Figure S3. (B) CCV fractions were prepared from cells incubated in either normal medium or in medium containing 189 μM copper (Cu). Both copper-ATPases are lost from the CCV fraction as early as 10 min after copper addition.

To investigate how the AP-1 knocksideways affects the steady-state distribution of some of the CCV cargo proteins, we mixed together control and knocksideways cells, then treated the cells with rapamycin for 1 hr. Immunofluorescence revealed that in the control cells, KIAA0319L, syntaxin 16, syntaxin 10, and ATP7A all had a juxtanuclear pattern, colocalizing strongly with the TGN marker TGN46 (Figures 4A; Figure S3; see also [26]). However, the knocksideways caused this pattern to become more diffuse and to spread out toward the cell periphery, similar to what we showed for the cation-independent mannose 6-phosphate receptor (CIMPR) in our previous knocksideways study [2]. This finding provides further support for a role for AP-1 in retrograde trafficking back to the TGN.

Because the trafficking of ATP7A and ATP7B is regulated by copper levels, we investigated whether copper affects the packaging of the two ATPases into CCVs. Cells were incubated for either 10 min or 1 hr in normal or high-copper medium, then CCV fractions were prepared. Even after 10 min, the high copper caused a striking depletion of both ATP7A and ATP7B from CCVs (Figure 4B). These observations, together with the knocksideways data, suggest that normally AP-1 continually retrieves ATP7A and ATP7B back to the TGN but that high copper levels prevent the two copper-ATPases from interacting with AP-1, possibly by inducing a conformational change that masks their dileucine sorting signals.

### Conclusions

By combining the knocksideways technique with CCV profiling, we have been able to gain unprecedented insights into the functional relationship between AP-1 and GGAs. Our results indicate that AP-1 acts as a linchpin in the formation of intracellular CCVs, and that if AP-1 is acutely lost, these vesicles cannot form. Unexpectedly, one of the CCV components that was most strongly dependent on AP-1 was GGA2. This result challenges the conclusions of a recent study carried out on yeast, which proposed that GGAs and AP-1 nucleate the formation of different populations of CCVs [11], but it is consistent with immunoelectron microscopy studies showing that at least some clathrin-coated-budding profiles are positive for both AP-1 and GGAs [9, 10].

Putting all of the available data together, our new results, together with reports in the literature, are most consistent with a role for AP-1 in bidirectional trafficking. We propose that GGAs act together with AP-1 to transport hydrolase-receptor complexes in the anterograde direction, from a juxtanuclear TGN/post-TGN compartment to a more peripheral endosomal compartment, where the hydrolases dissociate. We also propose that AP-1 has a second role in retrieving ligand-free receptors, together with proteins like KIAA0319L, ATP7A and ATP7B, and various SNAREs, from the peripheral endosomal compartment back to the juxtanuclear TGN/post-TGN compartment. Further evidence supporting a role for AP-1 in endosome-to-TGN trafficking comes from the finding that endocytosed anti-CIMPR remains in a peripheral compartment in AP-1 knocksideways cells but moves to a juxtanuclear compartment in controls [2]. In addition, immunolocalization studies have shown that GGAs are mainly associated with the TGN, whereas much of the AP-1 is associated with tubular endosomes [27, 28]. Why the two adaptors localize, at least in part, to different compartments is not clear, but differences in the molecular makeup of the compartments (e.g., ARF concentration, phosphoinositide composition) probably contribute.

From the present study, we can conclude that the AP-1 pathway (although not necessarily AP-1 itself) sorts a variety of biologically important transmembrane and lumenal proteins. These include not only hydrolases and their receptors but also multiple SNAREs and a number of proteins that are mutated in human genetic disorders. In the case of ATP7A and ATP7B, AP-1-mediated trafficking is probably essential for normal function, and it will be important to determine whether the same is true for other cargo proteins.

Whereas the AP-1 knocksideways produced global effects on intracellular CCVs, the GGA2 knocksideways affected only a single accessory protein, the three hydrolase receptors, and a range of hydrolases and other proteins residing in the lysosomal lumen. This indicates that the major role of GGA2 in HeLa cells is to transport lysosomal proteins in the anterograde direction. Other GGAs may be more important for trafficking of other types of cargo, such as ubiquitinated proteins [29], especially because GGA2 cannot completely compensate for loss of GGA1/GGA3 in knockout mice [15].

Although the GGAs have been relatively well characterized, we still know virtually nothing about the functions of most of the other peripheral membrane proteins associated with intracellular CCVs. However, by analogy with endocytic CCVs, we suspect that many of them will turn out to be alternative adaptors for cargo proteins whose sorting signals cannot bind directly to AP-1. By extending the knocksideways approach to some of the CCV “accessory factors,” we hope to be able to assign functions to proteins that until now have remained elusive.

## Supplemental Information

Document S1. Figures S1–S3 and Supplemental Experimental Procedures
