# Supplementary material for: Distinct and Overlapping Roles for AP-1 and GGAs Revealed by the “Knocksideways” System
Source: Curr Biol. 2012 Sep 25;22(18):1711–6. doi: 10.1016/j.cub.2012.07.012 (PMC3485558; doi:10.1016/j.cub.2012.07.012)
Supplement: Document S1. Figures S1–S3 and Supplemental Experimental Procedures [file mmc1.pdf]

Current Biology, Volume 22

Supplemental Information

Distinct and Overlapping Roles

for AP-1 and GGAs Revealed

by the “Knocksideways” System

Jennifer Hirst, Georg H.H. Borner, Robin Antrobus, Andrew A. Peden, Nicola A. Hodson, Daniela A. Sahlender, and Margaret S. Robinson

Supplemental Inventory

1. Supplemental Figures and Tables

Figure S1, related to Figure 1

Figure S2, related to Figure 3

Figure S3, related to Figure 4

Table S1, related to Figure 2 (see separate .xlsx document)

2. Supplemental Experimental Procedures

3. Supplemental References

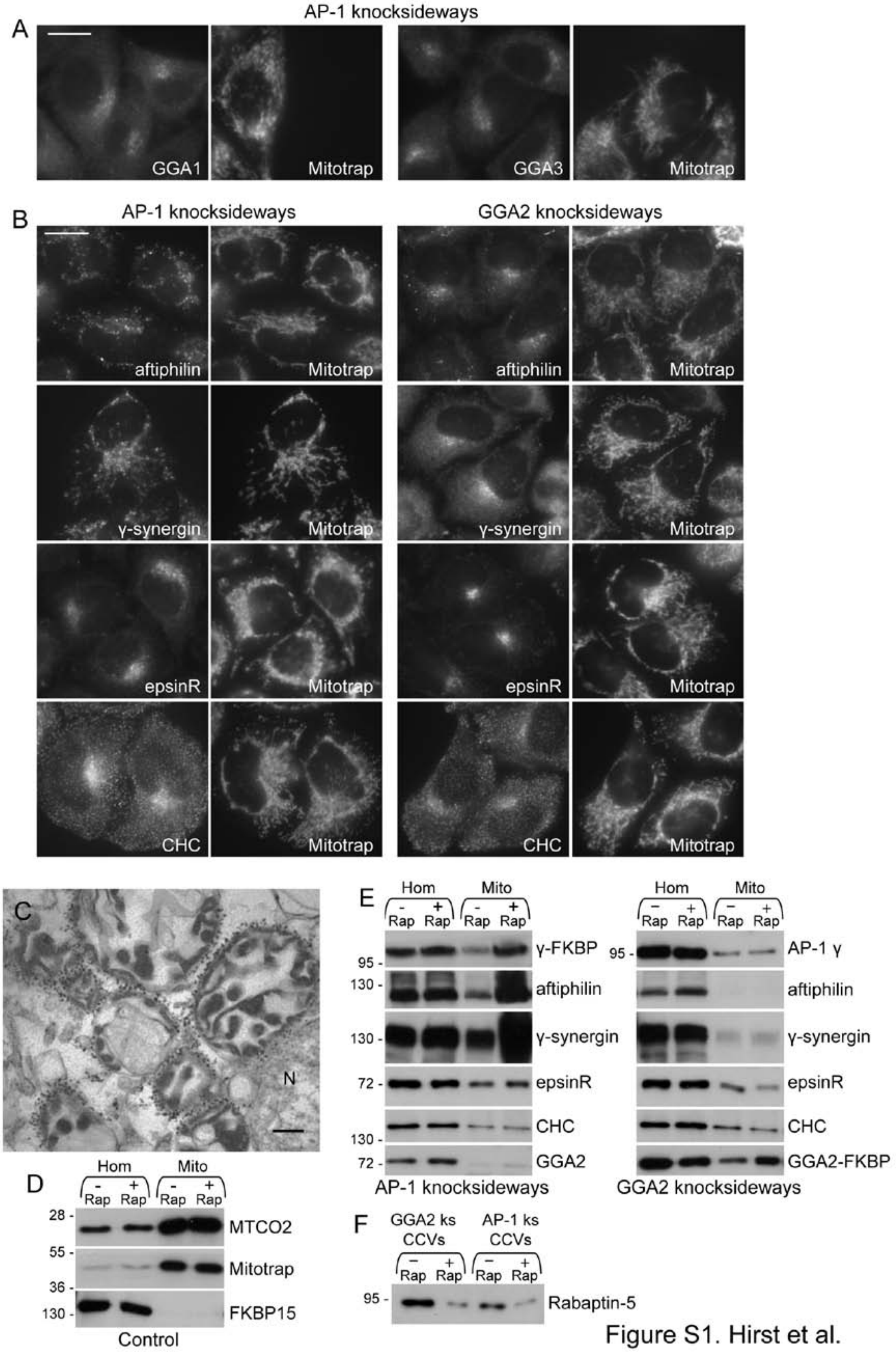

Figure S1. Hirst et al.

Figure S1. Behaviour of Various Coat Components in Knocksideways Cells, Related to Figure 1

(A) Immunofluorescence images showing that GGA1 and GGA3 did not relocate to mitochondria in AP-1 knocksideways cells. Scale bar: 20 μm.

(B) Immunofluorescence images showing that in the AP-1 knocksideways cells, aftiphilin and γ-synergin also relocated to mitochondria in the presence of rapamycin, while epsinR and clathrin did not. In the GGA2 knocksideways cells, none of these proteins relocated to mitochondria. Scale bar: 20 μm.

(C) Mitochondria were isolated using micro-magnetic beads (~10-20 nm) coated with an antibody recognising an outer mitochondria membrane protein. Electron microscopy showed that the major contaminants of the isolated mitochondria were nuclei (N). The magnetic beads can be seen as small electron-dense particles surrounding each mitochondrion. Scale bar: 200 nm.

(D) Western blot showing enrichment of the mitochondrial protein MTCO2 and of Mitotrap (labelled with anti-GFP) in the immuno-isolated mitochondria. There was no rerouting of the endogenous FKBP protein FKBP15, possibly because it does not cycle on and off membranes fast enough.

(E) Western blots showing that proteins that relocate to mitochondria by immunofluorescence are enriched in the immuno-isolated mitochondria after rapamycin treatment.

(F) Western blot showing that rabaptin-5 is lost from CCVs not only after a GGA2 knocksideways, but also after an AP-1 knocksideways, even though it was not detected by mass spectrometry. Apparent molecular weights of proteins are indicated.

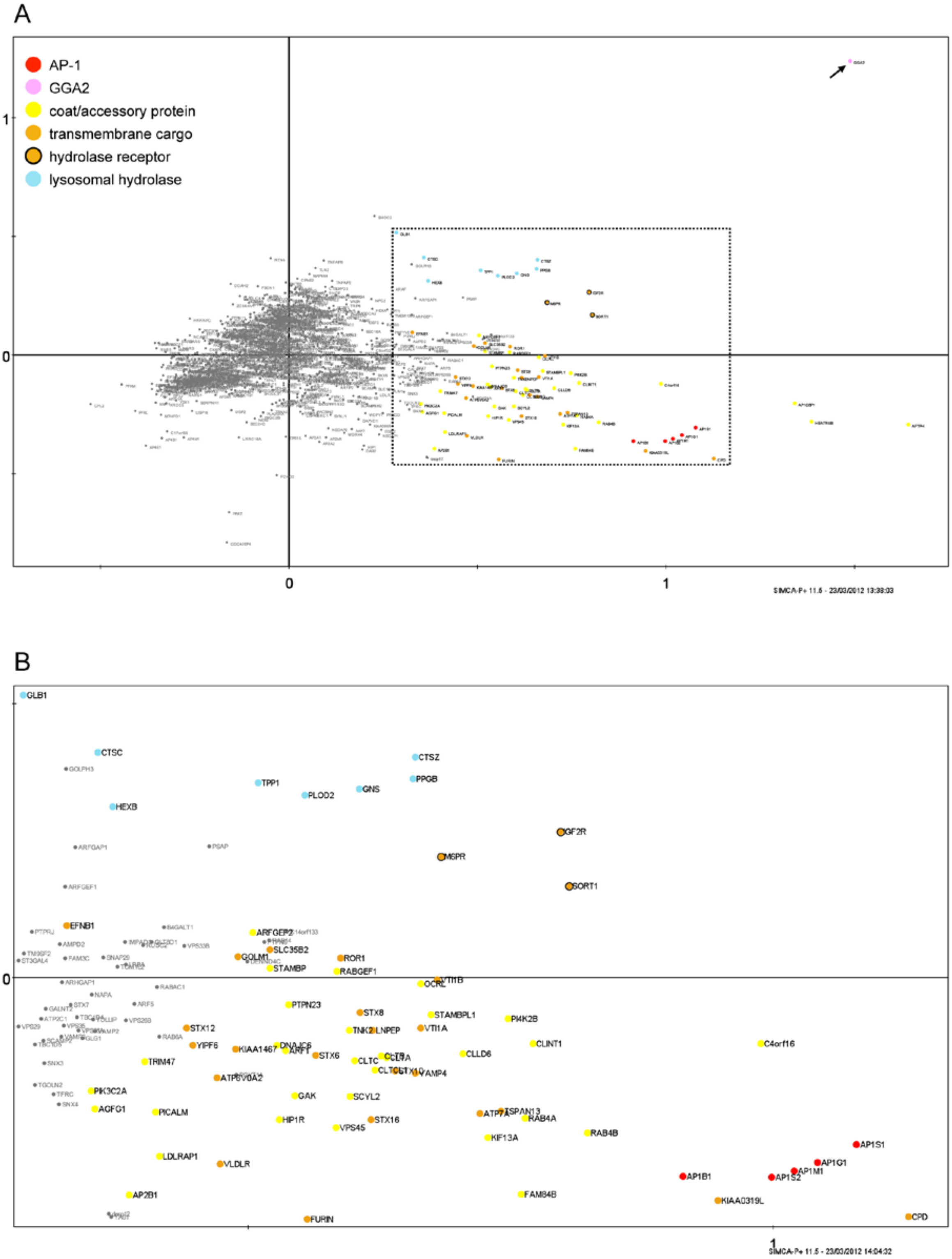

Figure S2. Hirst et al.

Figure S2. Principal Component Analysis of the SILAC Data, Related to Figure 3

(A) A principal component "profiling" analysis was carried out [17] to visualise the behavior of proteins across all SILAC datasets, without averaging repeat experiments. Diverse coat and cargo proteins cluster near AP-1. Hydrolases and their receptors also cluster, but are shifted towards GGA2. Only proteins identified in all four SILAC AP-1 or GGA knocksideways experiments were included in the profiling analysis. Proteins are annotated using UniProt names (http://www.uniprot.org/). The position of GGA2 is indicated (arrow).

(B) Magnified view of the section outlined in (A). Various coat and cargo proteins cluster with AP-1, indicating that these proteins are constituents of AP-1 positive CCVs. Lysosomal hydrolases and hydrolase receptors form distinct clusters that are shifted towards GGA2, which suggests that their incorporation into CCVs is (at least partially) GGA2-dependent.

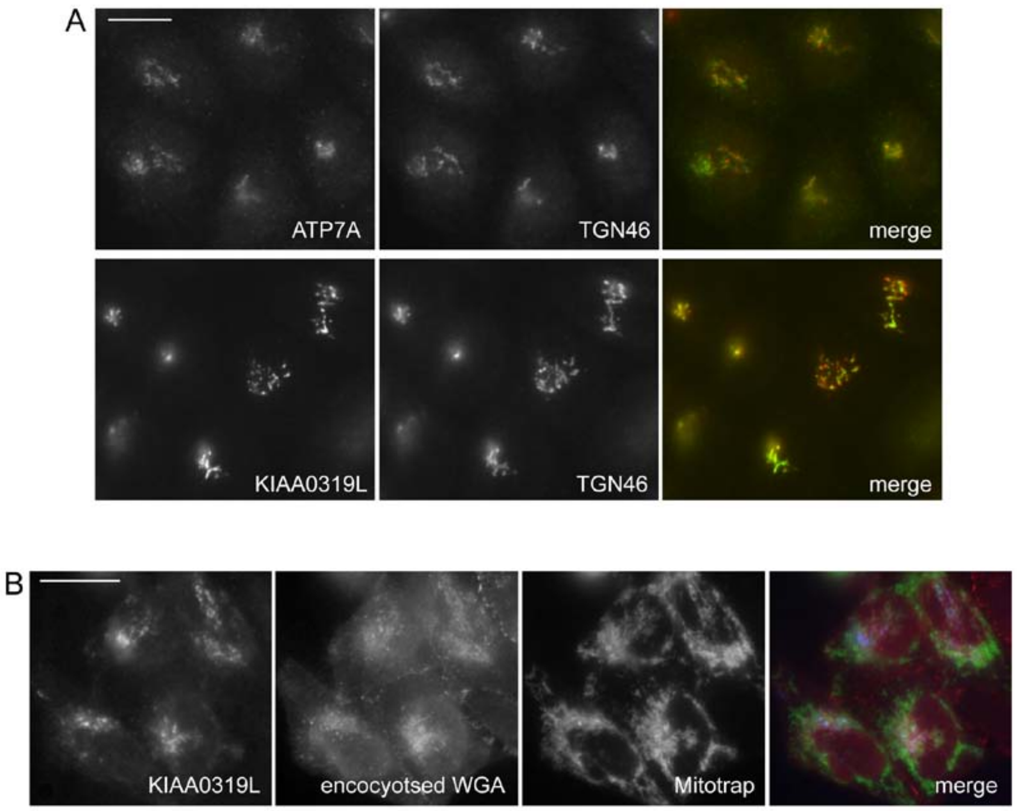

Figure S3. Additional Double Labelling for ATP7A and KIAA0319L, related to Figure 4

(A) Immunofluorescence images showing that under control conditions, both ATP7A and KIAA0319L show extensive overlap with TGN46.

(B) In AP-1 knocksideways cells, KIAA0319L is in the same vicinity as endocytosed AlexaFluor 680-conjugated wheat germ agglutinin (fed to the cells at a concentration of 2 μg/ml for 60 minutes), although the finer details are different. Scale bars: 20 μm.

Table S1. Complete Proteomic Profiling Data, Related to Figure 2

The table contains the complete proteomic data analyzed in this study, collated from five SILAC experiments (one control Mitotrap only, two AP-1 knocksideways and two GGA knocksideways). In each experiment, a CCV-enriched fraction was prepared from SILAC Heavy-labelled, rapamycin-treated cells, and quantitatively compared to SILAC Light-labelled, non-rapamycin-treated CCV fraction. In total, 2,511 proteins were identified in the CCV-enriched fraction by mass spectrometry. In all worksheets 'Gene Names' refer to a unique identifier (UniProt gene name; http://www.uniprot.org), and 'AP-1 ave' and 'GGA ave' refers to the average of the two biological repeats of the AP-1 and GGA2 knocksideways, respectively.

Worksheet 'MASTER data'

Raw data files were processed using MaxQuant and the primary output was a list of identified proteins, a ratio of relative abundance (Light/Heavy ratio=R, linearly normalised), and the number of quantification events (Count=C).

Worksheet 'FINAL data'

The raw data were processed to exclude entries that were only picked up in one experiment, had <5 quantification events in total, or were changed more than 1.5-fold in the control (Mitotrap only) experiment. The results were then ordered according to rank for either AP-1 knocksideways or GGA2 knocksideways from highest to lowest, with the number of entries reduced to 1,556 proteins for AP-1 knocksideways and 1,612 for GGA2 knocksideways. The entries were then colour coded according to known associations. The colours represent lysosomal hydrolases (blue), AP-1 subunits (red), GGA2 (pink), peripheral membrane proteins with potential association with AP-1 (yellow), integral membrane proteins with either known or candidate associations with AP-1 (orange). For proteins that were depleted 2-fold or more, double asterisks (**) were added to indicate that they were identified as likely CCV components by proteomics for the first time in the present study, and single asterisks (*) were added to indicate that they were identified as likely CCV components by proteomics for the first time in our recent profiling study [17] but not in previous proteomics studies. A small subset of the proteins depleted 2-fold or more are not shown in color because they are peripheral/cytosolic proteins with no previous associations with AP-1; however, some may prove in future to be genuine AP-1-associated proteins. In particular, C14orf133, Rab14, and Vps33B show similar behavior not only in the present study but also in our recent profiling paper [17], indicating that they function together, and all three have been associated with endosomal trafficking [S5].

Worksheet 'Lysosomal hydrolases'

Of the 23 lysosomal hydrolases identified in the GGA knocksideways, 15 of them and one other lumenal lysosomal protein (progranulin) were affected 2-fold or more, and are shown ranked from highest to lowest. Those indicated by # were not present in the final data set because <5 peptides were identified or they were only picked up in one experiment. These proteins are for reference only, although they follow the same trend as the other lysosomal hydrolases.

Worksheet 'Peripheral membrane proteins'

In total 49 peripheral membrane proteins were identified where potential associations with AP-1 have been published. Of these associations, 43 are validated here by being depleted at least 2-fold in the AP-1 knocksideways, or independently by co-immunoprecipitation (IP) using antibodies against AP-1 γ [S4] or GST-γ-ear pulldowns (PD), and ranked highest to lowest. Those indicated by # were not present in the final data set because <5 peptides were identified or they were only picked up in one experiment. They are listed for reference only,

but they were predicted to be AP-1/AP-2-associated in our profiling study [17]. For long-standing associations with AP-1 (e.g., the AP-1 subunits themselves), we have cited a single review article [S6].

Worksheet 'Integral membrane proteins'

In total 37 integral membrane proteins were identified that were affected at least 2-fold in an AP-1 knocksideways, and ranked highest to lowest. Over half of these represent known interactions, and the remaining represent candidates for new associations. The largest category by functional classification is SNAREs. Note that of the 37 integral membrane proteins affected in an AP-1 knocksideways, only the three lysosomal sorting receptors (IGF2R, M6PR and SORT1) are affected in the GGA2 knocksideways. Membrane protein topologies are as defined by Uniprot (http://www.uniprot.org).

Supplemental Experimental Procedures

Antibodies

Antibodies used in this study include in-house antibodies against AP-1 γ, aftiphilin, γ-synergin, epsinR, and clathrin [S1] and against the SNAREs syntaxin 10 and syntaxin 16 [S2]; commercial antibodies against GFP (3E6, MP Biomedicals), MTCO2 (ab3298, Abcam), KIAA0319L (ab105385, Abcam), ATP7A (sc-376467, Santa Cruz), ATP7B (ab124973, Abcam), and rabaptin-5 (A302-821A, Bethyl); and antibodies generously provided against FKBP15 and TGN46 (Matthew Seaman, CIMR, Cambridge, UK) and GGA2 (Doug Brooks, Women's and Children's Hospital, North Adelaide, Australia). HRP-labeled secondary antibodies were purchased from Sigma, and fluorescently labeled secondary antibodies from Invitrogen.

siRNA Knockdowns

Knockdowns were performed using the following On-Target Plus siRNA reagents from Dharmacon.

AP-1 γ (L-019183-02; GAAGAUAGAAUUCACCUUUUU) for knockdown of AP-1 in knocksideways experiments.

AP-1 γ (SMARTpool: L-019183-00) and AP-1 μ1 (SMARTpool; L-013196-00) for conventional knockdown in Figure 1C.

CLTC/CHC (SMARTpool; L-004001-01) for conventional knockdown in Figure 1C.

GGA1 (J-013694-08; CACAGGAGUGGGAGGCGAU)

GGA2 (J-012908-11; UGAAUUAUGUUUCGCAGAA)

GGA3 (J-012881-11; UGUGACAGCCUACGAUAAA) for knockdown of all three GGAs in knocksideways experiments.

Control knockdowns were performed with a non-targeting Smartpool siRNA (D-001810-10). Transfection of siRNA was achieved using Oligofectamine (Invitrogen) and Optimem, following the manufacturer's instructions. For immunofluorescence microscopy, a single-hit 72-h knockdown protocol was used, but for proteomics we used a single-hit 96-h protocol to reduce the impact of the unlabeled amino acids in Optimem. Knockdown efficiencies were determined by Western blotting and showed >85% depletion of the target proteins.

Tissue Culture and Immunofluorescence Microscopy

HeLaM cells [S3] were grown in Dulbecco's Modified Eagle's Medium (DMEM, Sigma) supplemented with 10% (v/v) foetal calf serum (Sigma), 2 mM L-glutamine, 50 units/ml penicillin, and 50 µg/ml streptomycin. The construction of the stable knocksideways cell lines is described below. For immunofluorescence microscopy, cells were plated into glass-bottom dishes (Mattek) and fixed with 3% formaldehyde followed by permeabilisation with 0.1% Triton X100. The cells were imaged with a Zeiss Axiovert 200 inverted microscope using a Zeiss Plan Achromat 63x oil immersion objective (NA 1.4), a Hamamatsu OCRA-ER2 camera, and IMPROVISION OPENLAB software.

For proteomics, cells were grown in SILAC medium supplemented with 10% (v/v) dialysed foetal calf serum (10,000 MW cut-off; Invitrogen), penicillin/streptomycin (Sigma) and an excess of L-proline (200 mg/L; Sigma), and either "Heavy" amino acids (L-arginine-$^{13}C_6{}^{15}N_4$:HCl (50mg/L) and L-lysine-$^{13}C_6{}^{15}N_2$:2HCl (100mg/L); Cambridge Isotope Laboratories), or the equivalent "Light" amino acids. Cells were grown for at least seven days to achieve metabolic labeling, and the average incorporation efficiency was approximately 95%, as determined by mass spectrometry. In all experiments, the cells to be treated with rapamycin were grown in Heavy SILAC medium.

Knocksideways

The construction of a stable cell line co-expressing the Mitotrap construct, consisting of a mitochondrial targeting signal, YFP, and an FRB domain, together with siRNA resistant AP-1 γ containing an FKBP domain in its hinge region, has been described in a previous paper [2]. For the present study, a control stable cell line expressing only Mitotrap was constructed, using the same MMLV-based vector, pQ(Mito-YFP-FRB), that was described in [2]. Cells were selected using hygromycin B, and FACS sorted for high levels of expression. The cells expressing Mitotrap only were used to control for spurious rerouting of proteins that contain FKBP domains and for non-specific rapamycin effects. They were also used as the cell line into which pLXIN(GGA2-FKBP) was transfected.

pLXIN(GGA2-FKBP) was constructed by amplifying the FKBP domain-coding sequence from the pLXIN(AP1γ-FKBP) plasmid by PCR, and adding BamH1 sites at either end. The PCR product was inserted into the hinge region of GGA2, and the coding sequence was then transferred into pLXIN between Sal1 and Not1 sites. In order to make this construct siRNA resistant, four silent mutations were introduced (TGAA**C**TA**C**GT**G**TC**C**CAGAA) into the siRNA-binding site using QuikChange Mutagenesis (Stratagene). Stable cells were selected with G418 (the medium also contained hygromycin to maintain expression of Mitotrap) and a clonal cell line was selected on the basis of expression levels of both Mitotrap and GGA2-FKBP. In particular, we wanted to ensure that the expression of Mitotrap was higher than that of GGA2-FKBP, so that it would not be saturated by GGA2-FKBP when rapamycin was added.

For the knocksideways experiments, cells were first treated with siRNAs targeting either AP-1 γ or all three GGAs to deplete the endogenous versions of the proteins (see above). The cells were then treated either with or without 200 ng/ml rapamycin at 37°C for various lengths of time. For experiments on isolated CCVs, the incubation time in rapamycin was 10 min. However, when the aim was to look for changes in the steady state localisation of CCV cargo proteins by immunofluorescence, the cells were incubated in rapamycin for 1 h, because only a small fraction of a particular cargo protein is present in CCVs at any one time, so it takes longer for a change in the overall localization of the protein to be detected. Rapamycin treatments were followed either by homogenization for CCV isolation, or by formaldehyde fixation for immunofluorescence microscopy.

Proteomics

Cells were grown in SILAC Heavy or SILAC Light medium and subjected to siRNA knockdown, followed by treatment of the SILAC Heavy cells with 200 ng/ml rapamycin for 10 min at 37°C. CCVs from the SILAC Heavy and SILAC Light cells were then isolated in parallel, maintaining rapamycin in the buffers where appropriate, and all preparations were performed at 4°C. Two confluent dishes (500 cm^2 each) of HeLa cells were scraped into ~5 ml buffer A (0.1 M MES, pH 6.5 (adjusted with NaOH), 0.2 mM EGTA, 0.5 mM $MgCl_2$). Cells were homogenized with a motorized Potter-Elvehjem homogenizer (20 strokes), and centrifuged at ~4,100 x g for 32 min. Supernatants were treated with ribonuclease A at 50 µg/ml for 30 min and then the membranes were pelleted by centrifugation at 50,000 rpm (135,700 x g RCF_{max}) for 30 min in a TLA-110 rotor (Beckman Coulter). Membranes were resuspended in ~300 μl buffer A using a 1 ml Dounce homogenizer, and mixed with an equal volume of 12.5% (w/v) Ficoll, 12.5% (w/v) sucrose, in buffer A. Samples were spun in a TLA 100.2 rotor at 20,000 rpm (21,700 x g RCF_{max}) for 25 min to pellet the bulk of the non-CCV membranes (pellet discarded). Supernatants were diluted with four volumes of buffer A, and centrifuged in a TLA-110 rotor at 40,000 rpm (86,900 x g RCF_{max}) for 30 min to obtain the CCV-enriched fraction. The yield of CCVs was between 30-50 μg as determined using a BCA protein reagent (Pierce). The SILAC Heavy and SILAC Light samples were mixed at equal protein concentrations with a maximum combined total of 50 µg, loaded in a single lane onto a preparative 1.5 mm 10% acrylamide gel, and run so that the sample separated

into a 2 cm strip. The gel was then washed, stained with Coomassie blue, and cut into 20 slices. Proteins were reduced, alkylated with iodoacetamide (A3221, Sigma), and in-gel digested with trypsin [S4], and the sample analysed by LC-MSMS in an Orbitrap mass spectrometer [17].

Data Analysis

For both the AP-1 knocksideways and the GGA2 knocksideways, datasets were produced of two independent biological repeats. In all cases the rapamycin-treated cells were those labeled with Heavy amino acids, and therefore the ratio data show fold depletion from CCVs. The raw data files were processed using MaxQuant, and the primary output for each SILAC comparison of CCVs was a list of identified proteins, a ratio of relative abundance (Light/Heavy ratio), and the number of quantification events (Count). Each MaxQuant output file was formatted in an identical manner to give a 'MASTER data' set (Table S1): (1) proteins with no gene names were removed; (2) where multiple entries existed, the entry with the highest number of counts was kept; (3) ratios were linearly normalised assuming equal protein quantities in both Heavy and Light samples (as in [17]). Then, the averages of the datasets for AP-1 or GGA2 knocksideways were determined, and only proteins that were identified in at least two experiments, and had a minimum number of 5 peptide counts, were kept in the dataset. A dataset was produced from a control cell line expressing only Mitotrap, and proteins that were affected >1.5 fold or <0.67 fold (see below) were removed from the AP-1 and GGA2 knocksideways datasets, resulting in a list of the complete 'FINAL data' used in this study (Table S1).

The control cell line expressed only Mitotrap, and CCVs were isolated with and without rapamycin treatment. These results were used to eliminate proteins from the AP-1 and GGA knocksideways datasets that were affected purely by the addition of rapamycin. Reassuringly, in these control cells there were very few proteins affected. The only proteins with >4 peptide counts that were affected 2-fold or more were four heterogeneous nuclear riboproteins, four DNA replication licensing factors, phospholipase A2 activation factor, dihydrolipoamide S-acetyltransferase, eukaryotic translation initiation factor 4H, and an unknown protein DKFZp781N1372. None of these proteins were significantly affected by the AP-1 or GGA2 knocksideways, and none of the proteins affected by the AP-1 or GGA2 knocksideways were significantly affected by rapamycin treatment in cells expressing Mitotrap only.

In our previous knocksideways study [2], we reported that the hydrolase DNase II was not affected in AP-1 knocksideways CCVs, based on Western blotting. However, the present study shows a 2.9-fold reduction in DNase II in AP-1 knocksideways CCVs, based on mass spectrometry. The reason for this discrepancy is most likely due to problems with the commercial antibody that we used to probe our Western blots in the previous study; in addition, mass spectrometry is much more quantitative than Western blotting, and the data on DNase II in the AP-1 knocksideways CCVs were very reproducible (2.97 and 2.78 in two separate biological repeats; see Table S1).

Statistical Analyses

For the profiling analysis in Fig. S2, SILAC ratio data were log transformed, centre scaled, and analysed by principal component analysis in SIMCA-P+ (Umetrics) [17].

Figure 2B shows the results of a non-paired T-test (two-tailed).

Mitochondrial Isolation

In order to isolate mitochondria, a standard isolation kit (Mitenyl) was used, following the manufacturer's instructions and with the supplied buffers, but with minor modifications. The protocol utilises small magnetic beads coated with an antibody to TOM-22, a mitochondrial outer membrane protein, to isolate mitochondria from whole cell lysates. Starting with $5x10^6$

cells, the cells were scraped off the dish with 1.5 ml lysis buffer (all buffers and incubations were carried out at 4°C) and homogenised by 18 strokes of syringe and needle (21 gauge). To reduce nuclear contamination, the lysate was spun at 100 g for 1 min, and then 1 ml of homogenate was mixed with 9 ml separation buffer and incubated with 50 µl of magnetic beads for 1 hr. The magnetic beads were collected by passing the sample through a column placed in a magnetic field, washed with 4 ml of separation buffer (repeated three times), and eluted in 1.5 ml separation buffer. The mitochondria were then concentrated into 100 µl storage buffer by spinning at 13,000 g for 2 min.

A sample of the isolated mitochondria preparation was fixed for ultrastructural analysis by the addition of freshly prepared 2% paraformaldehyde/ 2.5% gluteraldehyde in 0.1 M sodium cacodylate buffer, pH 7.3, for 1 hr at room temperature. The pellet was postfixed with 1% osmium tetroxide in 0.1 M sodium cacodylate buffer, pH 7.3, en bloc stained with 0.5% uranyl acetate in 0.05 M sodium maleate buffer pH 5.2 for 1 hr, dehydrated in ethanol, and embedded in Araldite CY212 epoxy resin (Agar Scientific). Ultrathin sections (60-70 nm) were stained with uranyl acetate and Reynolds lead citrate, and viewed in a transmission electron microscope (model CM 100; Philips). By electron microscopy, the purified mitochondrial fraction contained nuclear material as a major contaminant (see Figure S1C). Despite several attempts to reduce the nuclear contamination using more stringent washing steps or pre-spinning, we could not reduce the contamination without compromising the yield of mitochondria. Therefore this preparation was not sufficiently pure for sensitive proteomics, but it was suitable for Western blotting.

Supplemental References

S1. Borner, G.H.H., Harbour, M., Hester, S., Lilley, K.S., and Robinson, M.S. (2006). Comparative proteomics of clathrin-coated vesicles. J Cell Biol. *175*, 571-578.

S2. Gordon, D.E., Bond, L.M., Sahlender, D.A., and Peden, A.A. (2010). A targeted siRNA screen to identify SNAREs required for constitutive secretion in mammalian cells. Traffic *11*, 1191-1204.

S3. Tiwari, R.K., Kusari, J. and, Sen, G.C. (1987). Functional equivalents of interferon-mediated signals needed for induction of an mRNA can be generated by double-stranded RNA and growth factors. EMBO J. *6*, 3373-3378.

S4. Antrobus, R., and Borner, G.H.H. (2011). Improved elution conditions for native co-immunoprecipitation. PLoS One *23*, e18218.

S5. Cullinane, A.R., Straatman-Iwanowska, A., Zaucker, A., Wakabayashi, Y., Bruce, C.K., et al. (2010). Mutations in VIPAR cause an arthrogryposis, renal dysfunction and cholestasis syndrome phenotype with defects in epithelial polarization. Nat. Genet. *42*, 303-312.

S6. Robinson, M.S. (2004). Adaptable adaptors for coated vesicles. Trends Cell Biol. *12*, 695-704.

S7. Hirst, J., Borner, G.H.H., Harbour, M., and Robinson, M.S. (2005). The aftiphilin/p200/gamma-synergin complex. Mol. Biol. Cell. *16*, 2554-2565.

S8. Neubrand, V.E., Will, R.D., Möbius, W., Poustka, A., Wiemann, S., Schu, P., Dotti, C.G., Pepperkok, R., and Simpson, J.C. (2005). Gamma-BAR, a novel AP-1-interacting protein involved in post-Golgi trafficking. EMBO J. *24*, 1122-1133.

S9. Nakagawa, T., Setou, M., Seog, D., Ogasawara, K., Dohmae, N., Takio, K., and Hirokawa, N. (2000). A novel motor, KIF13A, transports mannose-6-phosphate receptor to plasma membrane through direct interaction with AP-1 complex. Cell *103*, 569-581.

S10. Delevoye, C., Hurbain, I., Tenza, D., Sibarita, J.B., Uzan-Gafsou, S., Ohno, H., Geerts, W.J., Verkleij, A.J., Salamero, J., Marks, M.S., and Raposo, G. (2009). AP-1 and KIF13A coordinate endosomal sorting and positioning during melanosome biogenesis. J. Cell Biol. *187*, 247-264.

S11. Kalthoff, C., Groos, S., Kohl, R., Mahrhold, S. and, Ungewickell, E.J. (2002). Clint: a novel clathrin-binding ENTH-domain protein at the Golgi. Mol. Biol. Cell *13*, 4060-4073.

S12. Hirst, J., Motley, A., Harasaki, K., Peak Chew, S.Y., and Robinson, M.S .(2003). EpsinR: an ENTH Domain-containing protein that interacts with AP-1. Mol. Biol. Cell *14*, 625-641.

S13. Mills, I.G., Praefcke, G.J., Vallis, Y., Peter, B.J., Olesen, L.E., Gallop, J.L., Butler, P.J., Evans, P.R., and McMahon, H.T. (2003). EpsinR: an AP1/clathrin interacting protein involved in vesicle trafficking. J. Cell Biol. *160*, 213-222.

S14. Borner, G.H.H., Rana, A.A., Forster, R., Harbour, M., Smith, J.C., and Robinson, M.S.(2007). CVAK104 is a novel regulator of clathrin-mediated SNARE sorting. Traffic *8*, 893-903.

S15. Zhang, C.X., Engqvist-Goldstein, A.E., Carreno, S., Owen, D.J., Smythe, E., and Drubin, D.J. (2005). Multiple roles for cyclin G-associated kinase in clathrin-mediated sorting events. Traffic *6*, 1103-1113.

S16. Crottet, P., Meyer, D.M., Rohrer, J., and Spiess, M. (2002). ARF1.GTP, tyrosine-based signals, and phosphatidylinositol 4,5-bisphosphate constitute a minimal machinery to recruit the AP-1 clathrin adaptor to membranes. Mol. Biol. Cell *13*, 3672-3682.

S17. Shinotsuka, C., Yoshida, Y., Kawamoto, K., Takatsu, H., and Nakayama, K. (2002). Overexpression of an ADP-ribosylation factor-guanine nucleotide exchange factor, BIG2, uncouples brefeldin A-induced adaptor protein-1 coat dissociation and membrane tubulation. J. Biol. Chem. *277*, 9468-9473.

S18. Shiba, Y., Takatsu, H., Shin, H.W., and Nakayama, K. (2002). gamma-Adaptin interacts directly with rabaptin-5 through its ear domain. J. Biochem. *131*, 327-336.

S19. Harasaki, K., Lubben, N.B., Harbour, M., Taylor, M.J., and Robinson, M.S. (2005). Sorting of major cargo glycoproteins into clathrin-coated vesicles. Traffic *6*, 1014-1026.

S20. Teuchert, M., Schäfer, W., Berghöfer, S., Hoflack, B., Klenk, H.D., and Garten, W. (1999). Sorting of furin at the trans-Golgi network. Interaction of the cytoplasmic tail sorting signals with AP-1 Golgi-specific assembly proteins. J. Biol. Chem. *274*, 8199-8207.

S21. Hirst, J., Miller, S.E., Taylor, M.J., von Mollard, G.F., and Robinson, M.S. (2004). EpsinR is an adaptor for the SNARE protein vti1b. Mol. Biol. Cell *15*, 5593-5602.
